# Block and Lock HIV Cure Strategies to Control the Latent Reservoir

**DOI:** 10.3389/fcimb.2020.00424

**Published:** 2020-08-14

**Authors:** Chantelle L. Ahlenstiel, Geoff Symonds, Stephen J. Kent, Anthony D. Kelleher

**Affiliations:** ^1^Kirby Institute, UNSW, Sydney, NSW, Australia; ^2^CSL Australia Ltd, Sydney, NSW, Australia; ^3^Department of Microbiology and Immunology, Peter Doherty Institute, The University of Melbourne, Melbourne, VIC, Australia; ^4^Melbourne Sexual Health Centre and Department of Infectious Diseases, Alfred Hospital and Central Clinical School, Monash University, Melbourne, VIC, Australia; ^5^ARC Centre for Excellence in Convergent Bio-Nano Science and Technology, The University of Melbourne, Parkville, VIC, Australia

**Keywords:** HIV-1, block and lock, cure strategies, epigenetic silencing, latent reservoir

## Abstract

The HIV latent reservoir represents the major challenge to cure development. Residing in resting CD4+ T cells and myeloid cells at multiple locations in the body, including sanctuary sites such as the brain, the latent reservoir is not eliminated by ART and has the ability to reactivate virus replication to pre-therapy levels when ART is ceased. There are four broad areas of HIV cure research. The only successful cure strategy, thus far, is stem cell transplantation using naturally HIV resistant CCR5Δ32 stem cells. A second potential cure approach uses gene editing technology, such as zinc-finger nucleases and CRISPR/Cas9. Another two cure strategies aim to control the HIV reservoir, with polar opposite concepts; The “shock and kill” approach, which aims to “shock” or reactivate the latent virus and then “kill” infected cells via targeted immune responses. Lastly, the “block and lock” approach, which aims to enhance the latent virus state by “blocking” HIV transcription and “locking” the HIV promoter in a deep latent state via epigenetic modifications. “Shock and kill” approaches are a major focus of cure studies, however we predict that the increased specificity of “block and lock” approaches will be required for the successful development of a sustained HIV clinical remission in the absence of ART. This review focuses on the current research of novel “block and lock” approaches being explored to generate an HIV cure via induction of epigenetic silencing. We will also discuss potential future therapeutic delivery and the challenges associated with progressing “block and lock” cure approaches as these move toward clinical trials.

## Introduction

**Figure 1:**
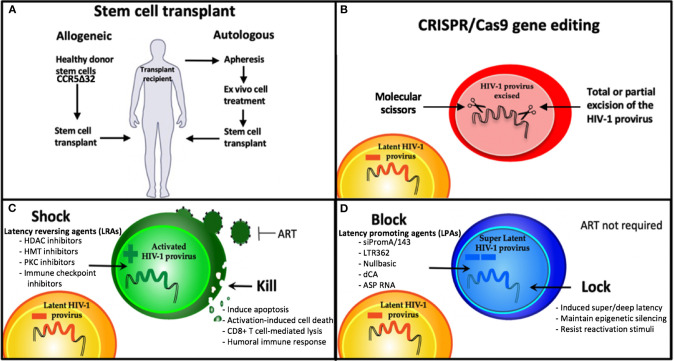
Strategies being developed for an HIV cure. **(A)** Stem cell therapies and **(B)** CRISPR gene therapy use modified cell therapies to target the latent reservoir. **(C)** Shock and Kill approach using latency reversing agents (LRAs) to eradicate the latent reservoir, and **(D)** Block and Lock approach using latency promoting agents (LPAs) to induce silencing of the latent reservoir to achieve sustained HIV remission, that is refractory to reactivation. HDAC, Histone deacetylase; HMT, histone methyl transferase; PKC, protein kinase C; dCA, didehydro-cortistatin A; ART, antiretroviral therapy.

Persistence of the HIV-1 latent reservoir is the major barrier to an HIV cure. Combined antiretroviral therapy (ART) is now such a highly efficacious treatment, that once a person living with HIV (PLWH) has commenced therapy and has an undetectable viral load they are then unable to transmit the virus (U=U) (Cohen et al., 2016; Bavinton et al., 2018; Rodger et al., 2019). However, ART interruption or cessation leads to a rapid rebound in viral load and significant morbidity. Therefore, treatment must be life-long. Hence, the aim of an HIV cure is to either (i) eradicate the latent reservoir from the body or (ii) have life-long remission of virus without the need for ART. Long-lived resting memory CD4+ T cells contribute a major component of the latent reservoir, followed by dendritic cells, macrophages, and microglial cells (Kumar et al., 2014; Kandathil et al., 2016; Honeycutt et al., 2017; Wallet et al., 2019). These cells types have a wide range of anatomical locations, including lymph nodes, gut-associated lymph tissue (GALT) (Yukl et al., 2013), liver (Penton and Blackard, 2014), genital tract (Cantero-Perez et al., 2019), and brain (Wallet et al., 2019). Some of these sites are further termed sanctuary sites, which are protected from ART penetration (i.e., the brain, testis, and lymph node B cell germinal centers) and pose additional challenges for HIV cure treatments (Eisele and Siliciano, 2012; Fletcher et al., 2014). Persistence of the latent reservoir occurs due to clonal expansion of infected cells and/or infection of long-lived reservoir cells (Chomont et al., 2009; Hiener et al., 2017; Lee et al., 2019). Many studies are attempting to characterize the HIV reservoir in order to understand the unique cell types and subsets involved (Hiener et al., 2017; Lee et al., 2019; Pardons et al., 2019; Horsburgh et al., 2020), and the provirus state; whether intact or defective (Bruner et al., 2016, 2019; Hiener et al., 2017), reactivatable, or non-reactivatable (Battivelli et al., 2018). Studies investigating the fundamentals of HIV persistence are vital to developing cure strategies. The current focus of HIV cure strategies can be broadly segmented into four main areas: 1. Cell/Gene therapy using stem cell transplantation, 2. Gene therapy via gene editing, 3. Shock and kill approaches, and 4. Block and lock approaches (Figure 1).

There are so far two examples of PLWH being successfully cured of HIV, with both cases utilizing cell therapy with stem cell transplantation, i.e., the Berlin and London patients (Hutter et al., 2009; Gupta et al., 2019). This approach employed naturally HIV-resistant donor CCR5delta32 stem cells for transplantation in patients being treated for associated malignancies i.e., undergoing myeloablative/chemotherapy treatments (Hutter et al., 2009; Gupta et al., 2019). While studies are ongoing to attempt to recapitulate this cure approach in other patients who also require stem cell transplants due to associated malignancies, this approach is not currently scalable nor feasible or desirable in the general population of PLWH who do not require such heroic treatment for malignant disease. This is due to the high risk involved in undertaking an allogeneic stem cell transplantation.

Gene therapy cure approaches also aim to eradicate the integrated latent reservoir and use a number of nuclease-mediated gene editing tools, i.e., molecular scissors, that cut genomic DNA in a highly specific manner. Some examples include clustered regularly interspaced short palindromic repeats (CRISPR)-associated protein 9 (CRISPR/Cas9) technologies (Kaminski et al., 2016a,b; Wang et al., 2016a; Miller et al., 2017; Dash et al., 2019), zinc finger nuclease (ZFN) (DiGiusto et al., 2016; Ji et al., 2018), and the transcription-activator-like effector nucleases (TALEN) (Shi et al., 2017) gene editing to excise the HIV-1 genome from the host genome. A benefit of these gene editing sequence approaches is the high specificity required to match the target sequence. However, this also means that due to the extreme sequence diversity present in the HIV-1 genome, a combination of multiple sequences will be required in order to ensure sequence diversity and the potential for future virus mutations in the target site is addressed. Additionally, off-target effects and virus escape have been reported (Wang et al., 2016b). Delivery of gene editing therapies to the target site are also a major challenge.

A further eradication cure approach is the commonly known “shock and kill” strategy, recently reviewed by Ait-Ammar et al. (2019). This approach aims to “shock” latently-infected cells into a reactivated state, using latency-reversing agents (LRAs) to activate virus transcription and then “kill” these reservoir cells via cytopathic effects, host immune responses or other targeted mechanisms. Several classes of LRAs have been investigated (Figure 1). One class of LRAs are epigenetic modifiers, such as histone deacetylase inhibitors (HDACi), histone methyltransferase inhibitors (HMTi), and DNA methyltransferase inhibitors (DNMTi), which all act on reversing the repressive epigenetic marks present in the HIV-1 promoter during latency. These LRAs result in global activation, relaxing epigenetic marks in not just the HIV-1 promoter, but any promoter that is epigenetically silenced by these mechanisms. Another challenge of LRAs is the variability of effect depending on the specific cell model (Spina et al., 2013). Successful reactivation by most LRAs *in vitro* have failed to induce sufficient reactivation to make a detectable impact on the HIV reservoir *in vivo* or *ex vivo* in patient latently-infected cells (Spina et al., 2013). Additional improvements in the ability to kill reactivated cells are also likely to be needed, such as a broadly neutralizing antibody (bNAb) PGT121 and a Toll-like receptor 7 (TLR7) agonist (Borducchi et al., 2018). Moreover, the shock and kill approach is not suitable for all cell types harboring latent virus, such as microglial cells in the brain, reviewed in Wallet et al. (2019). This is due to reactivation of microglial cell reservoirs resulting in neuroinflammation, a key component of HIV-associated neurocognitive disorders (HAND) (Wallet et al., 2019). As demonstrated by several groups, it is unlikely that targeting a single mechanism of HIV-1 latency will be sufficient to reactivate the majority of the virus reservoir (Jiang et al., 2015; Rochat et al., 2017; Das et al., 2018; Ait-Ammar et al., 2019). Instead, a combination of LRAs, targeting multiple mechanisms of HIV-1 latency is likely to be required for an effective sterilizing cure without ART.

A fundamentally different and potentially more realistic approach to reservoir control is known as “block and lock.” This functional cure strategy aims to permanently silence the latent reservoir using latency promoting agents (LPAs) to “block” virus transcription and “lock” the virus promoter in a latent state via repressive epigenetic modifications. Permanent control of the HIV-1 promoter means ART is no longer required. The block and lock approach mimics natural virus latency by inducing a state of latency, described recently by the terms “super latency” or “deep latency.” The features of HIV-1 transcription and the latency are described below, including the specific aspects mimicked by the block and lock approach. A precedent for forcing HIV-1 into a permanently silenced state via the block and lock epigenetic silencing approach has been set by the many ancient, epigenetically silenced human endogenous retroviruses (HERVs) that comprise ~8% of the human genome (Lander et al., 2001). This supports the feasibility and potential longevity of the block and lock approach. A major benefit of this cure approach, when mediated by RNA therapeutics, is that highly specific sequence targeting is required. Similar to CRISPR gene editing approaches, multiplexing of several RNA therapeutics targeting different sites in the virus genome will be necessary to address the global sequence diversity of HIV-1 (Ahlenstiel et al., 2015; Pang et al., 2018).

**Figure 2:**
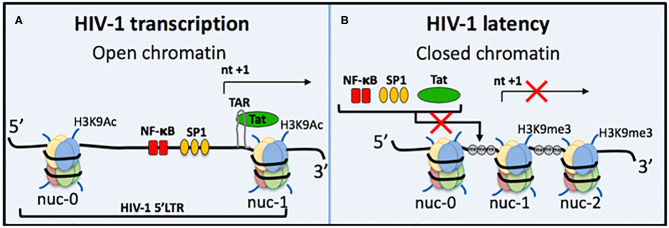
Regulation of HIV-1 transcriptional activity. **(A)** Active HIV-1 transcription occurs when chromatin is in an open structure, enabling important transcription factors to bind and activate virus transcription. These include NF-κB (red bars) and Sp1 (orange ovals). The TAR loop is also accessible for HIV-1 Tat protein to bind and further activate transcription. Active epigenetic marks, e.g., Histone 3 Lysine 9 Acetylation (H3K9Ac), are also present. **(B)** During HIV-1 latency several mechanisms can prevent virus transcription; (i) repositioning of nucleosomes causes chromatin compaction to form heterochromatin, (ii) heterochromatin occludes important transcription factors and Tat from binding, (iii) DNA methylation (gray circle, me) of CpG islands also prevents transcription, and (iv) histone post-translational modifications include increased repressive epigenetic marks, e.g., Histone 3 Lysine 9 trimethylation, and a decrease in active epigenetic marks, e.g., H3K9Ac. Some block and lock cure approaches mimic all four of these traits of HIV-1 latency, e.g., siRNA PromA.

Understanding the process of HIV-1 transcription and the molecular mechanisms involved in regulating HIV-1 latency is important for developing targeted therapies (Figure 2). A trademark feature of all retroviruses, including HIV-1, is integration of the viral genome into the host genome. Integration site selection is not random and can affect the transcriptional status depending on whether integration occurs in an active or silent gene. The process requires the HIV-1 protein integrase (IN) and the host protein Lens Epithelium-Derived Growth Factor (LEDGF/p75) [reviewed in Symons et al. (2018)]. Establishment and maintenance of integrated HIV-1 provirus in a range of latent reservoir cell types likely requires different latency molecular mechanisms. Transcriptional activity of the HIV-1 promoter, the 5′LTR, is regulated by multiple different factors. Autoregulation by the HIV-1 transactivator Tat protein is a major factor and results from Tat binding to the transactivation-responsive region (TAR), an RNA loop element located downstream of the transcription initiation start site located at nucleotides +1 to +59 (Figure 2A). Following Tat binding to TAR, the positive elongation factor, P-TEFb, is recruited to form a transcription complex, which results in Tat-mediated transactivation of transcription initiation and elongation via RNAPol II.

Host cellular transcription factors also play role in regulating HIV-1 transcription, such as NF-κB and Sp1, which are located between nucleosome (nuc)-0 and nuc-1 in the 5′LTR (Figure 2). NF-κB in particular is a major transactivator of virus transcription and has been specifically targeted by several RNA block and lock cure approaches. The chromatin environment is also a factor in HIV transcription regulation. Epigenetic silencing can reduce levels of NF-κB, which then change the efficiency of initiation and reduces Tat protein levels to result in transcription inhibition and the onset of HIV-1 latency. NF-κB is then required for virus reactivation by re-stimulating Tat production to restore transcription efficiency. Epigenetic silencing of the latent provirus can include histone post-translation modifications, such as histone methylation (Marban et al., 2007; Imai et al., 2010; Zhang et al., 2017), histone deacetylation (Verdin et al., 1993; Van Lint et al., 1996; Lusic et al., 2003), and crotonylation (Jiang et al., 2018) (Figure 2). Another characteristic of repressive epigenetic silencing in latent HIV-1 is DNA methylation of CpG islands (Kauder et al., 2009; Chavez et al., 2011).

## Block and Lock Strategies

### Epigenetic Silencing/Transcriptional Gene Silencing (TGS)

**Figure 3:**
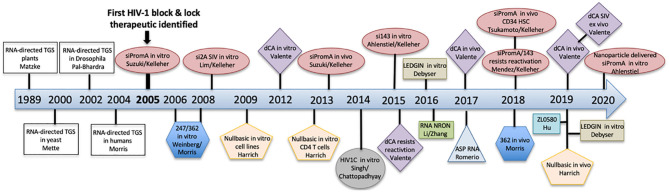
A timeline summarizing the discovery of transcriptional gene silencing and the development of block and lock HIV-1 therapeutics.

HIV cure strategies that follow the block and lock approach all have a common feature, which is the induction of epigenetic silencing or transcriptional gene silencing (TGS) in the HIV-1 promoter to suppress virus replication. The term epigenetics refers to heritable changes in gene expression that are independent of DNA sequence (Eccleston et al., 2007). Epigenetic silencing, or TGS, is a highly conserved process that was first discovered in plants, followed by studies showing existence of the pathway in *Caenorhabditis elegans*, Drosophila, yeast and finally in humans in 2004 pioneered by Morris et al. (2004) (Figure 3). Although the term block and lock has only been adopted in the last 4–5 years, studies extensively developing, identifying and characterizing block and lock HIV-1 therapeutics have been ongoing since the mid-2000s. Closely following on the discovery of transcriptional gene silencing in mammalian cells in 2004, was the first published “block and lock” HIV-1 study in 2005, which identified an HIV-1 promoter-targeted siRNA, known as siPromA (Figure 3). A comprehensive summary of block and lock HIV-1 cure/therapeutic development is described in Table 1. These block and lock therapeutics, while all inducing various degrees of epigenetic silencing, can be distinguished by effect longevity, i.e., whether the antiviral agent can only block HIV transcription while the treatment is given or if the treatment can maintain HIV latency following ART interruption.

**Table 1:** Summary of block and lock HIV-1 cure/therapeutic development.

**Block and lock therapeutic**	**Class**	**HIV-1 target**	**Stage**	**Sponsor/collaborator**	**References**
PromA	si/shRNA	Promoter, NF-kB sites	*In vivo*	Calimmune Inc.	Suzuki et al., 2005, 2008, 2011, 2013; Yamagishi et al., 2009; Ahlenstiel et al., 2012, 2015; Méndez et al., 2018; Tsukamoto et al., 2018a,b
143	si/shRNA	Promoter AP-1/COUP-TF Nuc-0	*In vitro*	University of New South Wales	Ahlenstiel et al., 2015; Méndez et al., 2018
LTR362as	si/shRNA	Promoter, NF-kB sites	*In vivo*	City of Hope	Weinberg et al., 2006; Turner et al., 2009, 2012; Zhou et al., 2018
ASP	HIV RNA	Promoter	*In vitro*	University of Maryland	Romerio et al., 2016; Zapata et al., 2017; Affram et al., 2019
LncRNA	lncRNA	Promoter	*In vitro*	City of Hope	Saayman et al., 2014
NRON	lncRNA	Tat	*In vitro* *Ex vivo*	Sun Yat-sen University	Li et al., 2016
Nullbasic	Small molecule inhibitor	Tat	*In vivo*	QIMR Berghofer Medical Research Institute	Meredith et al., 2009; Lin et al., 2012, 2014, 2015; Apolloni et al., 2013; Jin et al., 2016, 2019; Rustanti et al., 2017, 2018
dCA	Small molecule inhibitor	Tat	*In vivo*	The Scripps Research Institute	Mousseau et al., 2012, 2015, 2019; Kessing et al., 2017; Li et al., 2019; Mediouni et al., 2019a,b
LEDGIN	Small molecule inhibitor	Integrase	*In vitro*	Katholieke Universiteit, Leuven	Vranckx et al., 2016; Lampi et al., 2019; Vansant et al., 2019
BRD4-inhibitor ZL0580	Small molecule inhibitor	Tat	*In vitro* *Ex vivo*	University of Texas Medical Branch	Niu et al., 2019
Torin1, pp242	Small molecule inhibitor	mTor	*In vitro* *Ex vivo*		Besnard et al., 2016

### RNA-Directed Epigenetic Silencing

**Figure 4:**
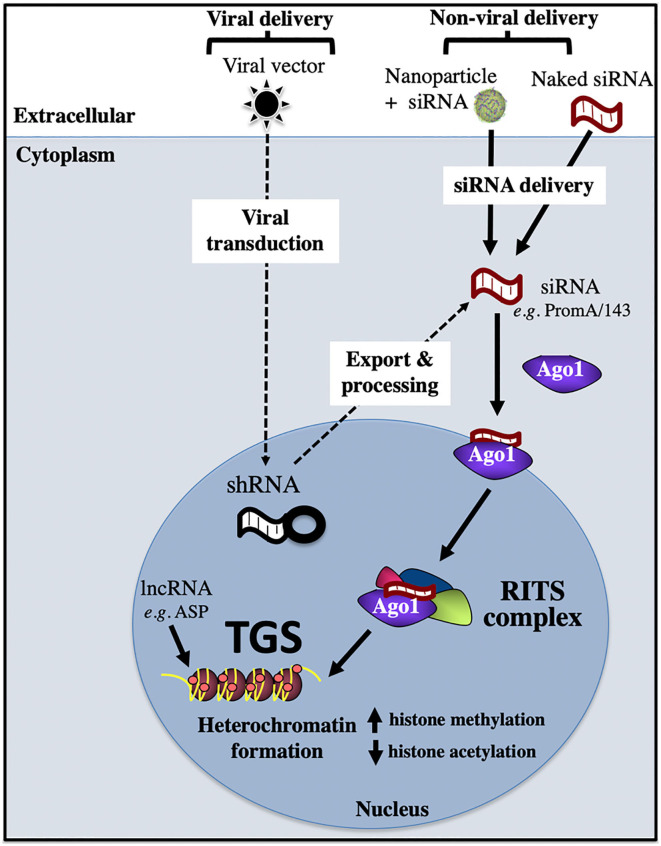
Transcriptional gene silencing (epigenetic silencing) pathway. Transcriptional gene silencing (TGS) can be mediated by viral or non-viral delivery of RNA sequences, which associate with the Argonaute protein, Ago1, then enter the nucleus to form the RITS complex, which recruits repressive epigenetic marks to induce chromatin compaction and silence gene expression. Ago1, Argonaute 1; shRNA, short hairpin RNA; RISC, RNA induced silencing complex; RITS, RNA induced transcriptional silencing complex; ASP, HIV-1 encoded antisense protein.

Epigenetic silencing can be induced by a range of RNA molecules, such as short interfering (si)RNA, short hairpin (sh)RNA, and long non-coding (lnc)RNA (Figure 4). In the case of siRNA, *in vitro* delivery is achieved via transfection reagents, e.g., lipofectamine, nucleofection, calcium phosphate; or cell penetrating nanoparticles that are loaded with siRNA. Once siRNA has been successfully delivered across the plasma membrane into the cytoplasm, it is then loaded onto the Argonaute 1 (Ago1) protein, with the 5′ siRNA region binding to the Ago1 PAZ domain and the 3′ siRNA end binding to the Ago1 MID domain. Subsequent transport of the siRNA/Ago1 complex into the nucleus is highly sequence dependent, as we have shown that only siRNA sequences that target complementary sites present in the host genome are able to be trafficked in the nucleus (Ahlenstiel et al., 2015). Once the siRNA/Ago1 complex has entered the nucleus, further proteins are recruited to form the RNA-Induced Transcriptional Silencing Complex (RITS), which results in repressive epigenetic marks being deposited on the promoter, such as increased histone and CpG methylation and decreased histone acetylation. Viral delivery of shRNA involves a cell being transduced with a viral vector expressing the shRNA of interest and entering the cell using a viral envelope (e.g., VSV-G). Nuclear delivery of shRNA then occurs, followed by export and processing into siRNA, where they follow the path outlined above (Figure 4). Novel RNAs that have been shown to induce the block and lock phenomena in HIV-1 are summarized below.

### HIV-1 Provirus-Targeted RNA: PromA/143

The first anti-HIV-1 therapeutic to induce epigenetic silencing via siRNA, consistent with the term “block and lock” was identified by the Kelleher laboratory in 2005, termed siPromA (Figure 3, Table 1). This specific RNA sequence targets the unique tandem NF-κB sites in the HIV-1 promoter to induce potent transcriptional gene silencing mediated by repressive epigenetic marks (Suzuki et al., 2005, 2008, 2011, 2013, 2015; Yamagishi et al., 2009; Ahlenstiel et al., 2012, 2015; Méndez et al., 2015, 2018). Due to the conserved sequence of the NF-κB transcription factor site, extensive studies have been performed to investigate the potential of this siRNA to induce off-target effects (Suzuki et al., 2011). However, since the sequence of the HIV encoded NF- κB binding sites is substantially different from those encoded by the host genome and as the 19 bp sequence of siPromA includes portions of both the tandem NF-κB sites and their linking sequence, a 19 bp sequence which is unique to the HIV-1 promoter and not found in the human genome sequence can be targeted with no identified off-target effects (Suzuki et al., 2011; Ahlenstiel et al., 2015). To demonstrate longevity of the silencing effect, *in vitro* studies in cell lines have shown a single siRNA dose was sufficient to suppress virus replication 1,000-fold for up to 15 days and for >1 year in cells stably expressing lentiviral shPromA (Suzuki et al., 2008; Ahlenstiel et al., 2015). The virus silencing effect induced by siPromA has also been reported in *in vitro* in PBMCs and monocyte-derived macrophages (Suzuki et al., 2008, 2013; Ahlenstiel et al., 2015).

A recent study has demonstrated that cells expressing promoter-targeted siPromA and/or si143 are robustly resistant to reactivation stimuli, with each siRNA sequence inducing a unique repressive epigenetic profile (Méndez et al., 2018). This supports the approach of multiplexing RNA sequences to both achieve enhanced virus latency and address the global diversity of HIV sequences. Characterization of siPromA has progressed into *in vivo* humanized mouse models, with a study in 2013 using PBMCs transduced with lentiviral vector expressing shPromA (Suzuki et al., 2013) and more recently a study using CD34+ HSCs transduced with lentiviral vector expressing shPromA (Tsukamoto et al., 2018a,b). Both studies showed that siPromA-induced silencing could provide protection against HIV-1 infection. These studies demonstrated significantly lower HIV-1 cell-associated RNA levels in siPromA-expressing CD4+ T cells isolated from blood and tissue (lymph nodes/spleen) and normal CD4:CD8 ratios when compared to controls. Current studies are investigating the viral rebound effect following withdrawal of ART treatment in humanized mouse models. Promising results indicate siPromA induces a delay in virus rebound post-ART interruption. A short, animated infographic describing the HIV-1 block and lock approach and the potential therapeutic use of siPromA is described in the following URL (https://kirby.unsw.edu.au/news/block-lock-pathway-hiv-remission).

### HIV-1 Provirus-Targeted RNA: LTR362

The next block and lock HIV-1 therapeutic identified was LTR362 in the Morris laboratory (Weinberg et al., 2006). This RNA sequence also targets the tandem NF-κB sites in the HIV-1 promoter and overlaps with 8 bp of the 19 bp siPromA sequence. A recent *in vivo* study in a humanized mouse model of HIV-1 infection investigated combining this RNA sequence with the gp120 A-1 aptamer and multiplexing with two post-transcriptional gene silencing (PTGS) RNAs targeting Tat and Rev mRNA (Zhou et al., 2018). The study demonstrated virus suppression and increased CD4+ T cells levels compared to controls. However, although LTR362 has been reported to induce epigenetic silencing through CpG methylation in the 5′LTR *in vitro* (Weinberg et al., 2006), this did not translate in the *in vivo* model, with protection against virus infection being attributed to the post-transcriptional control of Tat and Rev (Zhou et al., 2018). Further studies will be required *in vivo* to investigate the contribution of histone methylation in TGS-induced by LTR362. This study highlights the potential therapeutic advantage of multiplexing both TGS and PTGS RNA sequences.

### SIV Provirus-Targeted RNA: si2A

Investigations by the Kelleher laboratory targeting the SIV provirus identified the third block and lock RNA sequence, termed si2A (Lim et al., 2008). This study was motivated to enable confirmation of the TGS-inducing effect of siPromA in a non-human primate model. Given the substantial difference in the SIV and HIV-1 promoter sequences, a novel RNA target in the SIV promoter needed to be identified to enable future non-human primate studies. The RNA sequence si2A targets ~51 bp upstream of the SIV_mac_251 NF-κB site. Additional RNA sequences targeting the SIV_mac_251 promoter were also identified, termed SIV-S4a and SIV-S12, which are directly adjacent to the NF-kB site and transcriptional start site (TSS), respectively (Lim et al., 2008). The SIV-S4a and SIV-S12 RNA sequences also induced TGS of SIV, but not to the same degree as si2A (Lim et al., 2008).

### HIV-1 Provirus-Targeted RNA: S4

A further TGS-inducing siRNA sequence that also targets the NF-κB sequence in HIV-1 subtype C was identified in 2014, termed S4-siRNA (Singh et al., 2014). The Chattopadhyay laboratory designed S4-siRNA to specifically target subtype C, which is prevalent in nearly 50% of PLWH worldwide and commonly in countries with a high HIV prevalence, e.g., Southern Africa and India. This study demonstrated significantly decreased vRNA levels in TZM-bl cells transfected with S4-siRNA *in vitro* and *ex vivo* in human PBMCs transfected with S4-siRNA (Singh et al., 2014). TGS induction was confirmed using chromatin immunoprecipitation (ChIP) assay to demonstrate recruitment of repressive epigenetic marks, H3K27me3 and H3K9me2 (Singh et al., 2014). While this RNA has therapeutic potential specifically for HIV-1 subtype C, no other subtypes are targeted, as HIV subtype C has three NF-κB sites and the linking sequence is substantially different from other subtypes.

### HIV-1 Encoded Antisense Protein *ASP*

An HIV-1 encoded antisense protein that functions to promote virus latency was identified by the Romerio laboratory in 2016 and is termed *ASP* (Romerio et al., 2016). ASP RNA is derived from the 3′LTR and has been shown to recruit the polycomb group repressive complex 2 (PRC2) to the HIV-1 5′LTR promoter, resulting in repressive epigenetic modifications, i.e., increased H3K27me3 and reduced RNAPII occupancy at the promoter (Zapata et al., 2017). A recent study by Affram et al. demonstrated that ASP is located in the nucleus of latent myeloid and lymphoid cell lines but translocates to the cytoplasm and cell surface upon stimulation with PMA (Affram et al., 2019). Further, ASP co-localized with gp120 on the cell surface and was observed in cell-free HIV-1 particles. This study reported that ASP is an accessory protein incorporated into the surface of HIV-1 virions and has potential as a therapeutic target (Affram et al., 2019).

### HIV-1 Encoded Antisense Long Non-coding RNA

The first HIV-1 encoded long non-coding (lnc)RNA was identified in 2014 by the Morris laboratory (Saayman et al., 2014). The lncRNA was shown to induce HIV-1 transcriptional silencing *in vitro* via recruitment of a chromatin-remodeling complex, involving DNMT3a, EZH2, and HDAC-1, to the virus promoter (Saayman et al., 2014).

### Long Non-coding RNA NRON

In 2016 the Zhang laboratory identified a lncRNA, termed NRON, which was observed to be highly expressed in resting CD4+ T cells (Li et al., 2016). NRON suppresses virus transcription by inducing degradation of the transactivator protein Tat. This process occurs via NRON binding to Tat and then associating with the ubiquitin proteasome components CUL4B and PSMD11, resulting in Tat degradation. These *in vitro* studies indicated NRON plays a role in HIV-1 latency. Further *ex vivo* studies in resting CD4+ T cells isolated from patients on suppressive ART demonstrated that NRON used in combination with the LRA SAHA was able to reactivate the latent provirus and has potential as a new target for latency reversal (Li et al., 2016). In pursuit of latency reversal, two lncRNAs have also been identified to activate HIV-1 replication, such as lncRNA HEAL (Chao et al., 2019) and lncRNA MALAT1 (Qu et al., 2019).

### Inhibitors Targeting Tat

Tat is 14 kDa protein that is a potent activator of HIV gene expression and essential for RNA polymerase II (RNAPII) synthesis of full-length transcripts of integrated provirus (Sodroski et al., 1985). Transcriptional elongation from the HIV-1 promoter is dependent on the Tat-mediated association of the pTEFb (positive transcription elongation factor) complex and TAR (trans-activation response element) of the nascent viral RNA (Laspia et al., 1989). Due to the critical requirement of Tat for robust viral gene expression, it is a promising target in the development of HIV cure therapeutics.

### NullBasic

The Harrich laboratory identified NullBasic in 2009, which was the first Tat inhibitor to induce a block and lock HIV-1 silencing effect (Meredith et al., 2009). This 101 amino acid transdominant Tat mutant has an altered basic domain (amino acids 49–57) and replaces wild-type Tat with the amino acid sequence GGGGAGGG. Thus, the basic domain has been mutated, including the TAR-binding region, hence the name NullBasic. HIV-1 transcription is inhibited by NullBasic through competition with endogenous Tat, but also via inhibition of Rev-mediated transport of virus mRNA (Meredith et al., 2009). *In vitro* studies have reported that CD4+ T cells transduced with a retroviral vector expressing NullBasic showed suppression of virus transcription and replication (Jin et al., 2016). The mechanism was confirmed to be via TGS using ChIP assay to detect repressive epigenetic marks, i.e., reduced RNAPII occupancy at the promoter and decreased H3K9 acetylation (Jin et al., 2016). A recent *in vivo* study using retroviral vector delivery of NullBasic to primary human CD4+ T cells and engraftment in a NSG mouse model demonstrated undetectable viral RNA in plasma samples up to day 14 post-infection and significantly reduced viral RNA levels in tissue-derived CD4+ T cells (Jin et al., 2019). Although there was no difference in viral mRNA levels at later time points, there were increased levels of CD4+ T cells in NullBasic treated mice, suggesting a survival advantage (Jin et al., 2019). NullBasic shows potential as a gene therapy candidate and warrants further investigation to optimize the permanence of silencing.

### Didehydro-Cortistatin A (dCA)

Another small molecule inhibitor targeting Tat is the Cortistatin A (CA) analog, didehydro-Cortistatin A (dCA), identified by the Valente laboratory in 2012 (Mousseau et al., 2012). This molecule induces a block and lock HIV-1 silencing effect via Tat inhibition, specifically by binding to the RNA hairpin TAR-binding/basic domain of Tat (Mousseau et al., 2012). CA is a natural steroidal alkaloid derived from the marine sponge *Corticium simplex* (Aoki et al., 2006), however the chemical synthesis of dCA is more sustainable and cost-effective and is generated from the steroid prednisone (Shi et al., 2011). Initial studies in cell lines and primary cells confirmed a significant decrease in viral mRNA and capsid p24 antigen following dCA treatment. Treatment with dCA was demonstrated to block transcription initiation/elongation with a decrease in RNAPII associated with the HIV promoter in dCA treated HeLa-CD4 cells compared to controls (Mousseau et al., 2012). A further study showed dCA treatment is resistant to reactivation by various LRAs in both cell lines and primary CD4+ T cells (Mousseau et al., 2015). This small molecule inhibitor progressed to *in vivo* humanized BLT mouse studies in 2017, which demonstrated that dCA treatment delayed virus rebound until day 19 compared to day 10 in control mice (Kessing et al., 2017). The silencing mechanism was recently reported to include formation of heterochromatin in the 5′LTR, with the presence repressive epigenetic marks, including decreased H3 acetylation and reduced RNAPII occupancy at the promoter (Li et al., 2019). dCA is the only block and lock therapeutic, thus far, to progress to *ex vivo* non-human primate studies. A study in 2019 reported the dCA treatment inhibits reactivation of SIV in latently infected Hut78 cells, as well as *ex vivo* isolated primary CD4+ T cell isolated from SIV_mac_239-infected rhesus macaques (Mediouni et al., 2019b). This is encouraging data and, similar to NullBasic, dCA warrants further investigation in order to enhance the permanency of silencing. Another important avenue of investigation is whether the dCA resistant strains isolated *in vitro* (Mediouni et al., 2019b) are also present in PLWA globally (Rice, 2019).

### Small Molecule Inhibitors Targeting Integration

#### LEDGINs

First identified in 2010 by the Debyser laboratory, LEDGINs inhibit HIV-1 integration (Christ et al., 2010). The rational design of these small molecule inhibitors specifically targeted the interaction between LEDGF/p75 and HIV integrase, which is essential for virus integration via tethering of the pre-integration complex to chromatin (Christ et al., 2010). Interestingly, *in vitro* studies in cell lines and primary cells treated with LEDGINs during infection demonstrated provirus integrated into silent genes, which were subsequently resistant to reactivation by various LRAs (Vranckx et al., 2016; Vansant et al., 2019). This approach has potential for treatment during acute infection to reduce integration events and potentially during shock and kill reactivation via LRAs to prevent reseeding of the reservoir.

### Small Molecule Inhibitors Targeting Epigenetic Readers

#### ZL0580 Targeting BRD4

The epigenetic reader bromodomain and extraterminal (BET) family protein BRD4 is involved in regulation of HIV-1 transcription. Previous studies by the Verdin laboratory showed BRD4 can suppress HIV-1 transcription elongation specifically via competition with Tat for binding to pTERb/CDK9 (Bisgrove et al., 2007). Structure-guided drug design in the Hu laboratory recently identified the small molecule inhibitor ZL0580, which induces Tat inhibition via selective binding to BRD4 (Niu et al., 2019). Additionally, suppression of transcription elongation was reported and shown to be due to repressive epigenetic marks in the HIV-1 5′LTR (Niu et al., 2019). Importantly, combined treatment with ZL0580 and ART in *ex vivo* CD4+ T cells isolated from three ART suppressed HIV-infected participants reported accelerated HIV-1 suppression and delayed virus rebound by 8, 9, and 15 days, respectively, compared to ART alone (Niu et al., 2019). Similar to other small molecule inhibitors, further investigations are needed, with possible combination of molecules, to ensure permanent silencing.

### Small Molecule Inhibitors Targeting mTor

#### Torin1 and pp242

In 2016, the Verdin laboratory identified the mammalian target of rapamycin (mTOR) signaling pathway as an important modulator of HIV-1 latency (Besnard et al., 2016). Inhibition of mTOR by small molecules Torin1 and pp242 suppressed the reactivation of provirus in the Bcl-2 HIV latency primary cell model and *ex vivo* in CD4+ T cells isolated from ART suppressed HIV-1 infected participants (Besnard et al., 2016).

## Delivery of Block and Lock Therapeutics

**Figure 5:**
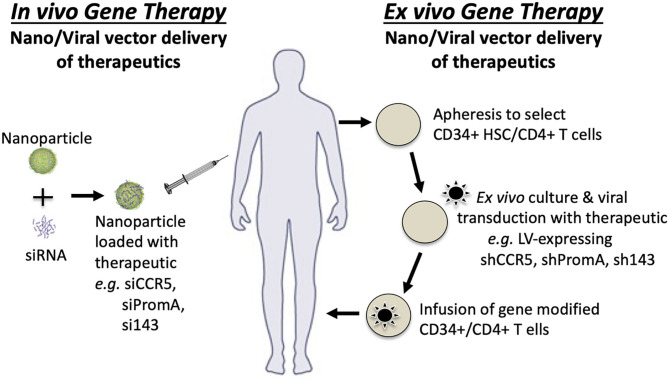
Gene therapy strategies for *in vivo* and *ex vivo* therapeutic delivery.

As described in Table 1, the development of several block and lock therapeutics has progressed to *in vivo* studies, primarily using humanized mouse models (Suzuki et al., 2013; Kessing et al., 2017; Tsukamoto et al., 2018a,b; Zhou et al., 2018; Jin et al., 2019) and one *ex vivo* study in non-human primates (Mediouni et al., 2019a). Delivery in these models is entirely dependent on the therapeutic class. RNA therapeutics have been delivered *ex vivo* to human CD34+ stem cells (Figure 5) or PBMCs using viral transduction of vectors expressing shRNA prior to infusion of modified stem or peripheral blood cells into the mouse. These studies have used a range of different humanized mouse models. The Tat small molecule inhibitor, dCA, has been orally delivered *in vivo* in a humanized mouse model. For cost effective and ease of treatment, direct *in vivo* delivery is preferable. An example of *in vivo* gene therapy currently being developed is nanoparticle delivery of the siPromA RNA therapeutic (Figure 5). Two of the biggest challenges for *in vivo* gene therapy to cure HIV, is (i) the widely distributed anatomical locations of the latent reservoir and (ii) the range of latently-infected cell types. Addressing both of these challenges will require *in vivo* gene therapies to be able to specifically target latently-infected cells, which is difficult as investigations to identify definitive latent reservoir specific biomarkers are ongoing (Fromentin et al., 2016; Descours et al., 2017; Sivro et al., 2018; Darcis et al., 2019; Pardons et al., 2019). An *in vivo* gene therapy that may inform HIV studies is being pioneered in the Peter-Kiem laboratory using foamy virus vector delivery of gene therapy for human X-linked severe combined immunodeficiency (SCID-X1) (Humbert et al., 2018). Foamy virus vectors have the specific advantage over VSV-G pseudotyped lentiviral vectors of being resistant to human serum inactivation, which is beneficial during *in vivo* delivery.

Although *in vivo* gene therapy is preferable, delivery of modified stem cells via viral vectors and *ex vivo* gene therapy has progressed into Phase I/II clinical trials. Targeting HIV-1 infection is the Cal-1 therapeutic lentiviral vector, which expresses shCCR5 and the fusion inhibitor C46, and has consistently demonstrated therapeutic benefits both *in vitro* and *in vivo* (Ringpis et al., 2012; Wolstein et al., 2014; Burke et al., 2015; Peterson et al., 2016; Symonds et al., 2016). The current clinical trial will provide data on reduced conditioning treatments that will guide future studies. Additionally, outside the field of HIV, *ex vivo* gene therapy is steadily improving, with multiple clinical trials studies currently underway in AAV-gene therapy for hemophilia A and B reviewed in Doshi and Arruda (2018).

## Conclusion

With the identification of the first block and lock HIV-1 therapeutic in 2005, the field has now started to rapidly expand the number of potential anti-HIV-1 therapeutics. These novel block and lock agents include RNA, transdominant protein, and small molecule inhibitors. Unlike a large number of “repurposed” shock and kill therapeutics, block and lock therapeutics are highly novel molecules, with no prior FDA approval and hence clinical trials of block and lock therapies have lagged behind. An advantage of the block and lock approach is the highly specific, HIV-1 targeted treatments in comparison to some non-specific shock and kill treatments e.g., histone deacetylase inhibitors that target global gene expression. A challenge of the block and lock approach, depending on the treatment e.g., siRNA, is the need to develop improved delivery systems that specifically target latent reservoir cells. As highlighted in this review, identification of potential block and lock therapeutics has increased dramatically in the last few years and studies in this area are steadily moving toward the clinical trials. Human trials will be necessary to assess whether this approach will ultimately help achieve a sustained HIV remission. Whether the best aspects of both cure approaches can be combined to potentially shock and kill latent reservoir cells that are able to be reactivated and then block and lock the remaining latent reservoir cells to ensure permanent HIV-1 latency is a combined approach that warrants future investigation.

## Author Contributions

CA and AK wrote the review. CA designed the figures and wrote the figure legends. GS and SK participated in the writing of the review. All authors contributed to the article and approved the submitted version.

## Conflict of Interest

GS was employed by the company CSL Australia, Ltd. CA, GS, and AK have a patent for siRNA sequences. The remaining author declares that the research was conducted in the absence of any commercial or financial relationships that could be construed as a potential conflict of interest. The handling editor declared a past co-authorship with one of the authors SK.
